# Perisaccadic Updating of Visual Representations and Attentional States: Linking Behavior and Neurophysiology

**DOI:** 10.3389/fnsys.2016.00003

**Published:** 2016-02-05

**Authors:** Alexandria C. Marino, James A. Mazer

**Affiliations:** ^1^Interdepartmental Neuroscience Program, Yale UniversityNew Haven, CT, USA; ^2^Medical Scientist Training Program, Yale University School of MedicineNew Haven, CT, USA; ^3^Department of Neurobiology, Yale University School of MedicineNew Haven, CT, USA; ^4^Department of Psychology, Yale UniversityNew Haven, CT, USA

**Keywords:** saccades, visual attention, neurophysiology, visual psychophysics, visual perception, receptive fields, receptive field remapping

## Abstract

During natural vision, saccadic eye movements lead to frequent retinal image changes that result in different neuronal subpopulations representing the same visual feature across fixations. Despite these potentially disruptive changes to the neural representation, our visual percept is remarkably stable. Visual receptive field remapping, characterized as an anticipatory shift in the position of a neuron’s spatial receptive field immediately before saccades, has been proposed as one possible neural substrate for visual stability. Many of the specific properties of remapping, e.g., the exact direction of remapping relative to the saccade vector and the precise mechanisms by which remapping could instantiate stability, remain a matter of debate. Recent studies have also shown that visual attention, like perception itself, can be sustained across saccades, suggesting that the attentional control system can also compensate for eye movements. Classical remapping could have an attentional component, or there could be a distinct attentional analog of visual remapping. At this time we do not yet fully understand how the stability of attentional representations relates to perisaccadic receptive field shifts. In this review, we develop a vocabulary for discussing perisaccadic shifts in receptive field location and perisaccadic shifts of attentional focus, review and synthesize behavioral and neurophysiological studies of perisaccadic perception and perisaccadic attention, and identify open questions that remain to be experimentally addressed.

## Introduction

Primates have evolved a sophisticated visual system to support active exploration of complex and often unpredictable dynamic natural environments. At the earliest stages of cortical processing, complex visual scenes are broken down into smaller, spatially localized features, e.g., edge contours of a particular orientation, contrast and color (Field, 1987). The spatially localized feature representation instantiated in primary visual cortex is substantially more compact than the raw retinal input (Barlow et al., 1964; reviewed in Simoncelli and Olshausen, 2001). However, even this highly compressed representation has the potential to overwhelm the brain’s computational capacity. To cope with this flood of information, primates and other vertebrates use a variety of selection mechanisms to prioritize processing of incoming sensory signals. Key among these are selective foveation, where the high acuity portion of the retina is directed toward salient scene features, and covert attention, where processing is enhanced at peripheral visual field locations without direct foveation. Both foveation and covert attention function to direct limited neural resources towards behaviorally relevant scene features. Understanding how eye movements and covert attention operate and interact during visual exploration is a major area of research in modern visual neuroscience.

The fovea, where visual acuity is highest, is relatively small, so it “sees” only a fraction of the scene at any given time. High-speed ballistic eye movements, known as saccades, are used to serially deploy the fovea towards behaviorally relevant scene features. As a result of these saccades, which occur several times per second in humans and monkeys (Yarbus, 1967; Snodderly, 1987), the projection of the visual scene onto the retina changes frequently, even when the scene is stable. Since the majority of visual areas in the primate brain are retinotopically organized, particularly in early and intermediate visual cortex, the neural representation of the visual scene is also dynamic, with different neuronal populations encoding a given scene feature from fixation to fixation. Nonetheless, our visual percept is remarkably stable, and observers can track features and objects across saccades without conscious effort, perhaps using an active compensation mechanism as long ago suggested by von Holmholtz (1866). In recent years, the neural circuits underlying visual stability (or indeed the necessity of such circuits) have become the focus of extensive theoretical and experimental inquiry. A variety of mechanisms for stabilizing visual perception have been proposed, including explicit spatiotopic or environmental maps (reviewed in Burr and Morrone, 2012), distributed or implicit neural representations of external space based on a dynamic combination of eye position signals with retinotopic visual inputs (reviewed in Salinas and Abbott, 2001), neural “previewing” via perisaccadic remapping of neural receptive fields (RFs; reviewed in Hall and Colby, 2011), and even the idea that the visual system incorporates a “default” Bayesian prior of environmental stability, such that in the absence of contrary evidence, we perceive our surroundings as stable (reviewed in O’Regan, 1992). The degree to which these varied mechanisms are consistent with experimental data or mutually compatible remains a matter of debate.

In addition to foveation, many vertebrates, including primates, use visuospatial attention to prioritize processing of important visual features. Although attention is often deployed at the point of fixation, scenarios arise where attention must be deployed towards peripheral field locations without moving the eyes, a process known as *covert* attention. For example, in primates, attention is likely an important component of social interaction where direct eye contact may be threatening, overly revealing, or inappropriate (Emery, 2000; George and Conty, 2008). Covert attention can speed reaction times, enhance target detection and discrimination abilities, improve spatial resolution and contrast sensitivity, and increase the accuracy of responses to stimuli at the attended location (reviewed in Carrasco, 2011; Anton-Erxleben and Carrasco, 2013). Electrophysiological recordings from behaving monkeys have shown that attention modulates neuronal firing rates in many brain regions. Attending inside a neuron’s RF generally (but not always) increases stimulus-evoked responses (Moran and Desimone, 1985; Petersen et al., 1985; Haenny et al., 1988; Colby et al., 1996; Treue and Maunsell, 1996; Luck et al., 1997). Attention can also affect other aspects of neural activity; for example, it can modulate synchrony between action potentials and local field potential oscillations (Fries et al., 2001; Chalk et al., 2010) or alter the noise correlations across neurons (Cohen and Maunsell, 2009). In humans, attentional modulation of BOLD signaling is detectable as early as area V1 (Brefczynski and DeYoe, 1999; Somers et al., 1999; Buracas and Boynton, 2007), and both visually evoked event-related potentials (Mangun and Hillyard, 1991) and ongoing EEG oscillations (reviewed in Herrmann and Knight, 2001) are modulated by attention.

Both saccades and attention are likely used together whenever complex, visually guided motor actions are performed outside the lab, e.g., driving, social interactions and virtually any task requiring visually guided manual manipulation of objects (Hayhoe and Ballard, 2005; Johnson et al., 2014). This raises an interesting and unanswered question: if eye movements change which neurons represent attended visual features and objects, how does top-down attention “know” which neurons to target for modulation after each saccade? In the remainder of this report, we will try to link behavioral studies of perisaccadic perceptual and attentional stability with specific, physiologically identified neural circuits and mechanisms in an effort to identify important resolved and unresolved questions. Our goal is to assess the extent to which current experimental evidence supports the idea of a simple, unified model or framework that can account for the diversity of observed perisaccadic effects and to identify useful directions for future research.

## Mechanisms for Maintaining Visual Stability

While intuitively it seems that uncompensated saccades should lead to perceptual instability, this is not necessarily the case, and we must consider seriously whether a compensatory mechanisms is really required to explain perception. There are other situations in which subjective awareness does not match either veridical visual inputs or neural activity. Consider that we are generally not consciously aware of blur in the visual periphery due to reduced photoreceptor density (Jennings and Charman, 1981; Merigan and Katz, 1990). In addition, studies of change detection have shown a surprising lack of awareness about large visual scene changes that dramatically alter the pattern of neural activity in early visual areas (Rensink et al., 1997; Beck et al., 2001; reviewed in Simons and Rensink, 2005). Perhaps stability represents a default assumption for the visual system; that is, even in the face of frequent changes in the retinal image, the visual system maintains a strong presumption of stability in the absence of strong evidence to the contrary (MacKay, 1972; Deubel et al., 1996). The behavioral consequences of assumed stability may be minimal—given the short duration of saccades, the likelihood of object displacements occurring *exclusively* during a single saccade is vanishingly small, so outside the lab, pre- and postsaccadic image mismatches can safely be attributed to sensory or motor noise (Niemeier et al., 2003). A study by Bridgeman et al. (1975) provided evidence in favor of the default assumption of stability by showing that relatively large stimulus displacements often go undetected when they occur during saccades, a phenomenon now referred to as saccadic suppression of displacement (SSD).

However, there are situations in which failure to properly assign retinal motion to external factors could lead to disaster (e.g., driving), and there is experimental evidence observers can override any default assumption of stability. Subjects are more likely to detect transsaccadic object displacements when other signals, such as a post-saccadic blanks (Deubel et al., 1996; Gysen et al., 2002), stimulus form changes (Demeyer et al., 2010) or displacements orthogonal to the saccade vector (Wexler and Collins, 2014) provide additional evidence of a change. The observation that postsaccadic stimulus manipulations can reduce SSD effects suggests that information about stimulus displacement is always encoded but only consciously available when some other cue signals a scene change. Also consistent with this idea is the finding that while subjects may fail to detect displacements, they can still accurately reach to displaced targets (Prablanc and Martin, 1992), indicating that accurate, updated position information is available to the motor system. Taken together, these results suggest that even if the visual system relies on a default presumption of stability, information about image changes is always encoded and accessible under the right behavioral conditions.

So, this leaves us with the question of how the visual system separates retinal motion into those components caused by the saccade and those caused by environmental changes. Historically this question and, more generally, questions related to updating of position information between or across saccades, has been studied using the double-step saccade paradigm. In a typical double-step experiment, subjects execute two saccades sequentially to two targets presented transiently before initiation of the first saccade (Hallett and Lightstone, 1976a,b). Making an accurate second saccade requires either an adjustment of the neural representation of the second saccade target’s location to compensate for first saccade or a spatiotopic representation of saccade targets.

### Explicit Spatiotopic Representation

As noted above, one possibility is that double step saccades, and perceptual stability in general, are mediated by spatiotopically organized brain regions (see Box 1). This would require buffering retinotopic information from each fixational snapshot into the proper region of the spatiotopic map, thus “filling in” the map. However, while some studies have reported evidence of spatiotopic maps in human MT, MST, V6, and LO (McKyton and Zohary, 2007; d’Avossa et al., 2007; Crespi et al., 2011), other studies have failed to replicate these findings and concluded these areas, as well as V1-V7, PPA and FFA are all retinotopically organized (Gardner et al., 2008; Golomb and Kanwisher, 2012). In macaques, there is general agreement that early and intermediate cortical visual areas are retinotopically organized (Essen and Zeki, 1978), including area MT (Krekelberg et al., 2003; Hartmann et al., 2011) and prefrontal areas like the frontal and supplementary eye fields (FEF and SEF; Russo and Bruce, 1993; although see Schall, 1991). And while there is some evidence of spatiotopic coding (although not necessarily a spatiotopic map) in parietal areas VIP (Duhamel et al., 1997; Zhang et al., 2004) and V6 (Galletti et al., 1995), neurons with spatiotopic tuning in these areas are relatively rare (Chen et al., 2013, 2014).

Box 1 **Coordinate system terminology.**

It is convenient to use different reference frames to specify the position of features and objects in the environment depending on behavioral goals or task demands. Neurons in various brain regions have been shown to use different reference frames and there is even evidence that some neurons switch reference frames depending on what the subject is doing. The key references frames discussed here are:

*Retinotopic.* The origin of a retinotopic reference frame is typically the fixation point, or the location in visual field that projects onto the fovea. This means that with each eye movement, the retinotopic coordinates of fixed environmental features are likely to change. Neurons in almost all early visual brain regions, both cortical and subcortical, are retinotopic (Gardner et al., 2008; Golomb and Kanwisher, 2012); retinal photoreceptors project in a labeled line manner to higher neurons such that neurons respond to stimuli falling on a specific region of the retina and not a specific location in the environment. The ascending visual system generally preserves retinal topography, at least up to the level of extrastriate visual cortex. This means that the distribution of activity in early visual areas is retinotopically mapped, and local neighborhood relationships between nearby visual features are preserved in the spatial distribution of neural activity. Note that neurons can be individually retinotopic, i.e., encode information using a retinotopic reference frame, without necessarily being part of a retinotopic map.

*Craniotopic.* Craniotopic reference frames are centered on the head. This means that the craniotopic coordinates of a scene feature are independent of the position of the eyes in the orbits. Both craniotopic and retinotopic reference frames are part of a larger family of egocentric reference frames. Other egocentric frames commonly discussed in the literature include body- and hand-centered, which are useful for spatial navigation and eye-hand coordination respectively. Neurons in some brain areas, particularly parietal cortex, have been shown to encode motor plans using body- and limb-based reference frames (Colby and Goldberg, 1999; Bremner and Andersen, 2012).

*Spatiotopic.* Spatiotopic reference frames, also known as world-centered, allocentric or environmental, are those that localize environmental features independently of the observer’s position and orientation in the environment, just as latitude and longitude denote a specific position on the earth’s surface regardless of an observer’s position. Although there is limited evidence of spatiotopic maps in the brain (although see d’Avossa et al., 2007; Crespi et al., 2011), some neurons, e.g., hippocampal place cells (O’Keefe, 1979) have long been known to exhibit spatiotopic tuning properties even though they are not necessarily organized into a topographic map.

This review focuses primarily on differences between retinotopic and spatiotopic processing in the brain, specifically how spatiotopic information can be encoded and acted on given the fundamentally retinotopic nature of early visual processing. The signals necessary for this transformation are readily available in visual cortex and there is experimental evidence that this occurs in parietal regions like area 7a (Andersen and Mountcastle, 1983). For further details, see review by Cohen and Andersen (2002).

Given the limited evidence for permanent spatiotopic coding, how likely is it that double step saccades are mediated by neurons coding for spatiotopic locations? It is conceivable that the reference frame used by a given neuron or cortical area could change depending on the behavioral task, which might explain apparently inconsistent reports of spatiotopic coding: when performing a manual grasping task, cells in monkey parietal area 5a, which are typically eye-centered, can shift to a hand-centered frame of reference (Bremner and Andersen, 2014). It is also possible there are heterogeneous distributions of neurons with different reference frames in some areas. For example, depending on the location within FEF, neurons can exhibit either eye- or head-centered tuning (Monteon et al., 2013). Similarly, different recording locations within monkey areas 7a, LIP, MIP and SEF (Martinez-Trujillo et al., 2004; Mullette-Gillman et al., 2005; Park et al., 2006; Crowe et al., 2008), and even human IPS (Pertzov et al., 2011) have been shown to exhibit tuning in varying reference frames. However, the functional role of changes in reference frame and the responses of spatiotopically coded neurons has not been examined in the context of double-step saccades or other visual stability tasks.

### Implicit Spatiotopic Representation: Gain Fields

Distributed codes are an alternative method for representing information in a spatiotopic reference frame without either an explicit spatiotopic map or neurons with spatiotopic RFs. One type of distributed code is a planar gain field, where the responses to visual stimuli are modulated multiplicatively by gaze angle. Gain fields were first described in posterior parietal areas LIP and 7a of the monkey (Andersen and Mountcastle, 1983; Andersen et al., 1985, 1987) and were subsequently reported in V1, V2 (Trotter and Celebrini, 1999; Rosenbluth and Allman, 2002), V3a (Galletti and Battaglini, 1989), V4 (Bremmer, 2000; Rosenbluth and Allman, 2002), V6/PO (Galletti et al., 1995), V6A (Breveglieri et al., 2012), IT (Lehky et al., 2008), FEF (Cassanello and Ferrera, 2007), VIP (Bremmer et al., 1999), AIP and F5 (Lehmann and Scherberger, 2013), dorsal and ventral premotor areas (Boussaoud et al., 1993, 1998) as well as the superior colliculus (SC; Van Opstal et al., 1995; Campos et al., 2006) of the monkey. In humans, gain field modulation of BOLD responses to visual stimuli has been reported throughout occipital (DeSouza et al., 2002; Merriam et al., 2013) and parietal cortex (Balslev and Miall, 2008; Williams and Smith, 2010).

Gain fields constitute a distributed code because the spatiotopic position of a given stimulus is encoded in the pattern of activity across a population of neurons and cannot be reliably determined from the responses of a single neuron. The critical aspect of the planar gain field model is that it describes a fundamentally retinotopic map from which spatiotopic information can be extracted by examining the pattern of activity across the neuronal population. This could allow for spatiotopic behavior, perhaps including spatiotopic targeting of top-down attention, and is consistent with much of the psychophysical and physiological data reviewed in the sections below. It is interesting, however, that in LIP, gain field effects can lag up to 150 ms after the saccade, yet animals can accurately localize targets appearing before the establishment of postsaccadic gain field modulation, suggesting that gain fields *alone* are unlikely to mediate localization in a spatiotopic reference frame (Xu et al., 2012).

### Perisaccadic Updating of Retinotopic Maps

In the absence of or in addition to the use of spatiotopic signals to detect displacement during saccades, systematic updating of retinotopic signals at the time of saccades could help maintain accurate position information. Evidence of updating comes from single neuron recordings made from monkeys executing double step saccades. During single saccades, individual neurons in the SC respond when the upcoming saccade matches that particular neuron’s preferred saccade vector. Similarly, between the first and second saccades in double-step saccade trials, SC neurons correctly signal the vector of the second saccade in a retinotopic frame of reference, indicating that updating has taken place to account for the first saccade (Sparks and Porter, 1983). A similar pattern of updating has been observed in monkey FEF (Goldberg and Bruce, 1990) and LIP (Gnadt and Andersen, 1988) as well as human parietal cortex (Medendorp et al., 2003), and disruption or lesioning of parietal cortex impairs double-step performance in both humans (Heide et al., 1995; van Donkelaar and Müri, 2002; Morris et al., 2007) and monkeys (Li and Andersen, 2001), presumably by disrupting the updating process. Although this work proves that updating to account for saccades occurs, it does not clarify the updating process. The saccade direction and magnitude information required for accurate updating is likely a corollary discharge (CD) or efference copy of the oculomotor command (Sperry, 1950; Holst and Mittelstaedt, 1971; reviewed in Wurtz, 2008; Wurtz et al., 2011). Sommer and Wurtz (2004) demonstrated the CD signal is relayed from SC through the mediodorsal nucleus of the thalamus to FEF, and interruption of CD causes incorrect attribution of movement induced by eye movements to the outside world (Stevens et al., 1976; Ostendorf et al., 2010, 2013; Whitham et al., 2011).

**Figure 1:**
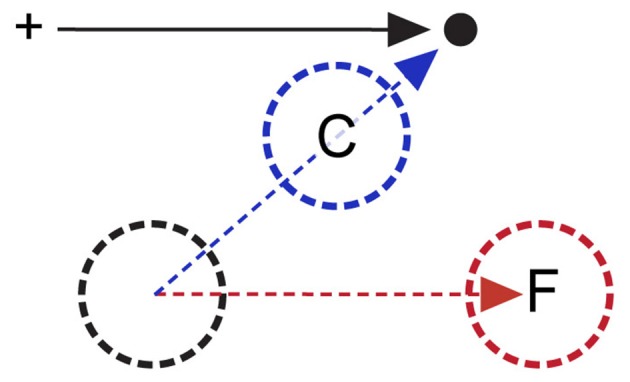
**Forward and convergent remapping.** Cross indicates initial fixation target; black dot and arrow indicate saccade target and vector respectively. Remapping occurs during the period between saccade target onset and saccade initiation, when the subject has planned but not yet executed the saccade. Black circle indicates a remapping neuron’s classical RF prior to instantiation of any saccade plan. The red circle (F) indicates forward remapping location (i.e., the “future field, FF”), in which the RF is shifted parallel to the impending saccade vector prior to initiation of the saccade indicated by the black arrow. The blue circle (C) reflects compressive remapping, in which the RF shifts towards the saccade endpoint (see text for more details).

Duhamel et al. (1992) reported that a subset of LIP neurons exhibit anticipatory RF shifts prior to saccades. These shifts translate the RF by the upcoming saccade vector. This type of predictive spatial shift in a fundamentally retinotopic map, which we will refer to as *forward remapping*, could provide continuity between pre- and postsaccadic visual representations since the shifts move RF locations to what will be their postsaccadic location (or “future field, FF”). Forward remapping has now been demonstrated in many areas (Walker et al., 1995; Umeno and Goldberg, 2001; Nakamura and Colby, 2002). However, studies using more comprehensive RF mapping techniques have suggested that at least in V4 (Tolias et al., 2001) and FEF (Zirnsak et al., 2014), RFs shift towards saccade endpoints rather than towards the FF location, a type of remapping that we will term *convergent remapping*. The distinction between forward and convergent remapping is illustrated in Figure 1. Several groups have described physiological signs of remapping in human visual areas using fMRI and EEG (Merriam et al., 2003, 2007; Parks and Corballis, 2008, 2010). However, due to either the limited spatial resolution of human imaging or constraints imposed by task design, it is impossible to distinguish between forward and convergent remapping in these studies. Whether or how perisaccadic RF shifts play a role in updating of retinotopic maps is not well understood. The distinction between forward and convergent remapping is further addressed below.

In all likelihood, perceptual stability is instantiated by a combination of neural mechanisms. For example, there is behavioral evidence that a weighted combination of CD, proprioceptive feedback and retinal input can be used to localize stimuli across saccades, depending on context (Poletti et al., 2013; Ostendorf and Dolan, 2015), and proprioceptive information is particularly useful in relative darkness, when retinal input is less informative (Gauthier et al., 1990). Successful computational models of perisaccadic visual perception incorporate visual input, CD, and proprioception (Ziesche and Hamker, 2011). In addition, while planar gain fields provide a computational algorithm for computing the spatiotopic position of features in the visual field, they are fundamentally retinotopic and still require some sort of remapping or updating mechanism to sustain a spatiotopic attentional locus. Although it may be possible to isolate individual mechanisms in the lab, full understanding of the maintenance of perceptual stability will depend on characterizing both individual mechanisms and their interactions.

## Reference Frames for Visual Processing

**Figure 2:**
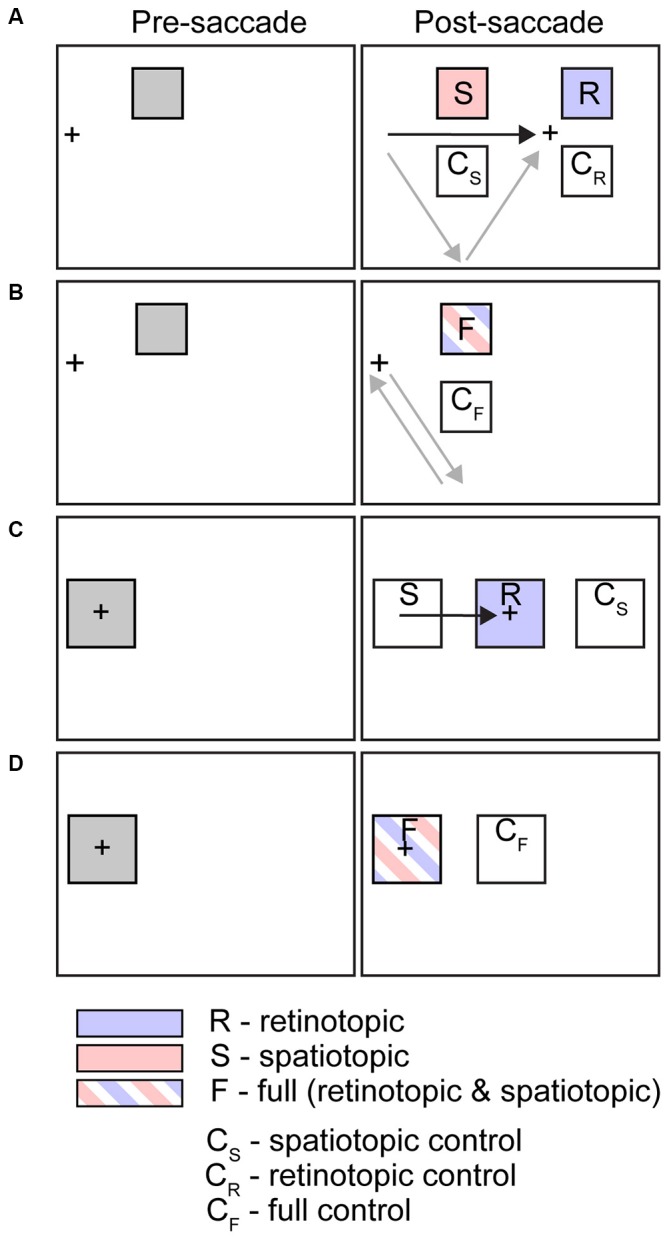
**Schematic illustrations of common experimental conditions used in studies of spatiotopic visual and attentional effects.** Figure shows stimulus configurations for tasks listed in Tables 1–3 and discussed in text. Left column indicates initial cue or adaptor location (presaccade, in trials that include a saccade); right column indicates target or test location (postsaccade, in trials that include a saccade). Arrows indicate saccade vectors. In some paradigms subjects execute a single saccade (black arrow); on others a sequence of saccades (gray arrows). **(A)** Saccade trials, peripheral stimuli. **(B)** Fixation (or saccade out and back) trials, peripheral stimuli. **(C)** Saccade trials, foveal stimuli. **(D)** Fixation trials, foveal stimuli.

Numerous studies have sought to identify the key reference frames underlying various psychophysical phenomena, including inhibition of return (IOR) and visual adaptation aftereffects. Many of these studies share a common design: an “event” transpires (stimulus appears, adaptation occurs, etc.) at one visual field location while subjects fixate, followed by saccade execution and then a psychophysical test at a new location corresponding to either the spatiotopic or retinotopic location of the original event or at a control location. Figure 2 schematizes some of the most common experimental designs from the literature.

### Inhibition of Return (IOR)

Posner and Cohen (1984) were the first to note that while the initial effect of an attentional cue is facilitative, facilitation lasts only a few hundred milliseconds and is followed by a period of suppression, during which behavioral responses to targets appearing at the cued location are actually impaired. This effect, known as inhibition of return (IOR), generally occurs for stimuli appearing at the spatiotopic location of the cue after a saccade (Posner and Cohen, 1984; Maylor and Hockey, 1985; Klein and Macinnes, 1999; Sapir et al., 2004; Mathôt and Theeuwes, 2010b; Pertzov et al., 2010; van Koningsbruggen et al., 2010; Hilchey et al., 2012; Satel et al., 2012; Krüger and Hunt, 2013), and this is consistent with the putative functional role of IOR in preventing attended items from being processed multiple times (Klein, 2000). In the first ~100 ms after saccades, IOR-like effects are detectable at both retinotopic and spatiotopic cue locations (Klein and Macinnes, 1999; Mathôt and Theeuwes, 2010b; Pertzov et al., 2010; Hilchey et al., 2012), suggesting that IOR is instantiated in a retinotopic area and actively updated around the time of the saccade (Table 1 summarizes many critical IOR reference frame studies). This is similar to sustained spatiotopic attention, which also appears to undergo perisaccadic updating, although there is a key difference: in the case of sustained attention updating is task-dependent, while for IOR updating appears to be obligatory (Golomb et al., 2008). Parietal cortex is necessary for IOR updating—TMS applied to either the superior parietal lobule (Sapir et al., 2004) or area AIPCx (van Koningsbruggen et al., 2010) abolishes spatiotopic, but not retinotopic, IOR. While it perhaps not surprising that parietal cortex is necessary for IOR updating, it is important to note that IOR updating is one of few spatiotopic phenomena shown to occur automatically in response to saccades. As such, localization to parietal areas could hint that parietal cortex is critical for perisaccadic updating in general, and that disabling these areas may also affect the updating of attention.

**Table 1:** **Perisaccadic updating of inhibition of return (IOR)**.

Reference	Conditions	Locations showing IOR	Target onset re: saccade (ms)	Task (response type)
Posner and Cohen (1984)	S, C_S_	S (R not tested)	600–1500*	Detection (key press)^†^
Maylor and Hockey (1985)	S, R, C_S_, C_R_	S	0–1000*	Detection (key press)
Klein and Macinnes (1999)	S, R, C_S_, C_R_	S and R	20	2AFC (saccade)^†^
Sapir et al. (2004)	S, R, C_S_, C_R_, F, C_F_	S and R	0–1500*	Detection (key press)
van Koningsbruggen et al. (2010)	S, C_S_	S and R	0–1400*	Detection (key press)
Mathôt and Theeuwes (2010b)	S, R, C_S_, C_R_	Early R, late S	50–1000	2AFC (saccade)
Pertzov et al. (2010)	S, R, C_S_, C_R_	S and R	0–600	2AFC (saccade)
Hilchey et al. (2012)	S, R, C_S_, C_R_	S and R	~40	2AFC (saccade)
Satel et al. (2012)	S, R, C_S_, C_R_	S and R	0–1200*	Detection (key press)
Krüger and Hunt (2013)	S, R, C_S_, C_R_, F, C_F_	S and R	0–1000*	Detection (key press)

In these IOR studies target stimuli appear eccentrically displaced from the fixation point and RT is used as the dependent measure. S, spatiotopic IOR; R, retinotopic IOR; NAFC, N alternative forced choice. *Indicates experiment was not gaze contingent, so it’s difficult to know exactly when test stimuli appeared relative to saccades. ^†^indicates a saccade target is treated as a cued location. Other abbreviations as in Figure 2.

### Adaptation

Visual adaption, in which prolonged exposure to one visual stimulus alters perception of subsequent stimuli, has been used extensively to study perisaccadic updating. The general approach is to adapt at one visual field location and then to compare adaptation state at the retinotopic and spatiotopic locations of the adapting stimulus after an eye movement. For example, in a recent study of tilt adaptation, observers fixated while an adapting tilted Gabor was presented peripherally, executed a saccade, and then indicated whether a test Gabor appearing at one of several locations was tilted clockwise or counter-clockwise (Zimmermann et al., 2013b). Studies of tilt adaptation have reported postsaccadic adaptation at retinotopic (Nishida et al., 2003; Knapen et al., 2010; Mathôt and Theeuwes, 2013), spatiotopic (Melcher, 2005, 2009) or both locations (Zimmermann et al., 2013b). Similarly conflicting responses have also been reported for face adaptation (Melcher, 2005; Afraz and Cavanagh, 2009; Wolfe and Whitney, 2015). The reference frame for motion adaptation appears to depend on the specific stimulus: adaptation to drifting Gabor patterns occurs only at the retinotopic location (Nishida et al., 2003; Wenderoth and Wiese, 2008; Knapen et al., 2009; Turi and Burr, 2012) while adaptation to random dot kinetograms and other types of motion has been reported at both retinotopic and spatiotopic locations (Turi and Burr, 2012; Morgan, 2014; Yoshimoto et al., 2014b). Recent studies of both figural and motion adaptation are summarized in Tables 2, 3, respectively.

**Table 2:** **Summary of perisaccadic updating of figural aftereffect studies**.

Reference	Type of aftereffect	Tested locations	Retinal eccentricity	Location of aftereffect	Test onset re: saccade (ms)	Behavioral task
Nishida et al. (2003)	Tilt	2F, 2R	Foveal	R (S not tested)	0–3000*	2AFC
Melcher (2005)	Tilt	XF, F	Peripheral	S (R not tested)	0–800*	2AFC
		S, C_S_	Foveal
Melcher (2009)	Tilt	F, Sx	Peripheral	S (R not tested)	0–800*	2AFC
Knapen et al. (2010)	Tilt	2F, 2C_F_, 2S, 2R	Peripheral	R	0–1250*	2AFC
Mathôt and Theeuwes (2013)	Tilt	R, S, C_R_, C_S_	Peripheral	R	100, 500	2AFC
Zimmermann et al. (2013b)	Tilt	F, S, R, C_S_	Peripheral	R and late S	0–300	2AFC
Nishida et al. (2003)	Contrast	2F, 2R	Foveal	R (S not tested)	0–3000*	2AFC
Melcher (2005)	Contrast	XF, F	Peripheral	No S (R not tested)	0–800*	Detection
		S, C_S_	Foveal
Nieman et al. (2005)	Color	2F, 2R, 2C_S_	Foveal	R (S not tested)	0–1000*	2AFC
Wittenberg et al. (2008)	Color	S, C_S_	Peripheral	S (R not tested)	20, 40	2AFC
Melcher (2005)	Face	XF, F	Peripheral	S (R not tested)	0–800*	3AFC (report face identify)
		S, C_S_	Foveal
Afraz and Cavanagh (2009)	Face	XF, F	Peripheral	No S (R not tested)	0–800*	2AFC (report gender)
		S, C_S_	Foveal
Wolfe and Whitney (2015)	Face	C_F_, S, R	Foveal	Upright: R and S	100	2AFC (report emotion)
				Inverted: R only
Nishida et al. (2003)	Spatial frequency	2F, 2R	Foveal	R (S not tested)	0–3000*	2AFC
Melcher (2005)	Pattern	XF, F	Peripheral	S (R not tested)	0–800*	2AFC
		S, C_S_	Foveal			
Nieman et al. (2005)	Depth	2F, 2R, 2C_S_	Foveal	R and C_S_	0–1000*	2AFC

Eccentricity indicates the visual field location of the adapting stimulus, which can be foveal or peripheral (displaced from the fovea). L, left; R, right; NS, nonspecific; horz, horizontal. A “2” prefixing an experimental condition indicates that 2 saccades were made; an “X” indicates that no presaccadic adapter was shown. * and Other abbreviations as in Figure 2 and Table 1.

**Table 3:** **Perisaccadic updating of motion aftereffects**.

Reference	Aftereffect (adaptor stimulus)	Tested locations	Retinal eccentricity	Aftereffect locations	Test onset re:saccade	Behavioral task
Mayhew (1973)	Rotation (2D grid)	R	Foveal	R (S not tested)	Always present	3AFC (left, right, no motion)
Nishida et al. (2003)	Motion direction (Gabor)	2F, 2R	Foveal	R (S not tested)	0–3000 ms*	Motion detection
Wenderoth and Wiese (2008)	Motion direction (RDK or Gabor)	F, 2F, C_F_, S, R, C_S_, C_R_	Peripheral	R and weak S	Not specified	2AFC
Ezzati et al. (2008)	Motion direction (RDK)	S, R, unmatched control	Foveal	R and S	0–500 ms*	2AFC
Knapen et al. (2009)	Motion direction (Gabor)	2F, 2C_F_, S, R	Peripheral	R	0–1250 ms*	2AFC
Turi and Burr (2012)	Motion direction (Gabor)	F, R, S, unmatched control	Peripheral	R	0–500 ms*	2AFC
Turi and Burr (2012)	Position (Gabor)	F, R, S, unmatched control	Peripheral	R and S	500 ms*	2AFC
Morgan (2014)	Relative position (Gabor)	R, S	Foveal	R	0 ms	2AFC
Yoshimoto et al. (2014a); Yoshimoto et al. (2014b)	Motion direction (Sine grating)	F, C_F_, R, S	Peripheral	Neg. priming: R Pos. priming: S	0–3000 ms*	2AFC

RDK, random dot kinetograph; Gabor indicates a drifting 2D Gabor pattern. “Unmatched control” indicates that the control location was not eccentricity matched to either the spatiotopic or retinotopic location. * and Other abbreviations as in Figure 2 and Tables 1, 2.

On face, there appears to be substantial heterogeneity and even apparent conflict in the literature regarding the specific reference frames for adaptation. However, upon closer examination, it is possible that much of this variability could be related to differences in stimuli and/or experimental methods. For example, which brain regions participate in a given task may vary in response to subtle changes to the task or even individual strategies. Likewise, some tasks may lead to automatic perisaccadic updating, while others may not, which could determine whether or not spatiotopic adaptation is observed. Another important consideration for comparing studies of adaptation is the control or baseline condition; some studies incorporate a spatially nonspecific (i.e., adaptation that generalizes to the entire visual display) and/or a full adaptation condition (i.e., adapt and test at the same location without any intervening eye movements), but others do not. It can be difficult to interpret adaptation effects without a suitable reference measurement. There is some evidence that saccades alone can affect adaptation state (Wenderoth and Wiese, 2008), even when there is no change in retinal stimulation, making the use of no-saccade trials a possibly problematic control condition. An elegant solution to this problem is to equate the number of saccades executed in every condition by having subjects always make two saccades, sometimes returning to the initial fixation point and sometimes saccading onwards to a new fixation point (Nieman et al., 2005; Knapen et al., 2010). Additionally, in some cases task geometry results in a lack of eccentricity-matched control locations for spatiotopic or retinotopic test stimuli. Another concern is that in some studies test locations can also be saccade targets, which are known to draw automatic attention (Hoffman and Subramaniam, 1995; Kowler et al., 1995; Deubel and Schneider, 1996), and this attention could modulate adaptation state directly. For example, Wolfe and Whitney (2015) reported spatiotopic face adaptation, but in their study, the spatiotopic but not the retinotopic test stimulus served as a saccade target, which means the spatiotopic, but not the retinotopic, stimulus was likely also the target of attentional facilitation (see “Attentional Facilitation at Saccade Endpoints” below). Indeed, Afraz and Cavanagh (2009) failed to find spatiotopic face adaptation when both the spatiotopic and retinotopic locations were displaced from the saccade endpoint to equate potential attentional contributions. Finally, although there is evidence that the precise timing of test presentation relative to saccades may influence whether adaptation effects are found in spatiotopic or retinotopic frames of reference (Zimmermann et al., 2013b), stimulus timing is variable across studies.

In short, the variability observed in published visual adaptation studies makes it difficult to draw definitive conclusions across studies. Moving forward, we hope to see studies carefully control the number of saccades and use peripheral, eccentricity-matched stimuli at retinotopic and spatiotopic test locations as well as control locations. Inclusion of both baseline and full adaptation conditions and careful control of stimulus presentation times relative to both saccades and the adaptation period would also facilitate cross-study comparisons. These steps would greatly facilitate identification of the general principles underlying peri- and transsaccadic visual adaption.

## Effects of Saccades on Visual Perception

Planning and executing saccades can impact visual perception, even in the absence of attention (to the extent that it is possible to dissociate attention from saccade targeting). In this section we focus on the proximal effects of saccades on accurate spatiotemporal localization of visual stimuli at both behavioral and neuronal levels.

### Perisaccadic Behavioral Findings

#### Spatial and Temporal Mislocalization

It has been known since the 1960s that observers systematically mislocalize stimuli that appear transiently around the time of saccade initiation (Matin and Pearce, 1965; Mackay, 1970; Morrone et al., 1997; Ross et al., 1997; reviewed in Ross et al., 2001; Matsumiya and Uchikawa, 2003). In human observers, saccades both translate the apparent stimulus location in the direction of the saccade and compress perceptual space towards the saccade endpoint. Although perisaccadic mislocalization is well studied, its functional significance remains a matter of debate. It has been suggested that mislocalization is an epiphenomenal consequence of timing mismatches between retinal and CD signals (Schlag and Schlag-Rey, 2002; Pola, 2004). Recently, Cicchini et al. (2013) suggested mislocalization could reflect an innate tolerance for displacement that facilitates transsaccadic object tracking by making it easier to group multiple features into a single object; however, it is not clear from this model why observers would experience spatial compression instead of simply reduced localization accuracy. In addition, there is some evidence that saccades are not even necessary for mislocalization errors. While saccade planning alone is insufficient to cause mislocalization (Atsma et al., 2014), simulated saccades during fixation (Mackay, 1970; Honda, 1995; Morrone et al., 1997; Ostendorf et al., 2006), visual masking (Zimmermann et al., 2013a) and spatial attention (Suzuki and Cavanagh, 1997; Liverence and Scholl, 2011) can all trigger mislocalization without saccades or saccade plans.

Saccades can also interfere with our perception of time; studies have shown both temporal compression (analogous to spatial compression) and temporal inversion effects, where the perceived temporal order of sequential stimuli is reversed (Yarrow et al., 2004, 2006; Morrone et al., 2005; reviewed in Yarrow, 2010). In general, the timing of temporal and spatial compression effects relative to saccades is similar (Binda et al., 2009). Temporal distortions are spatially localized (Knöll et al., 2013), are strongest near saccade endpoints (Georg and Lappe, 2007), and can also be triggered by simulated saccades (Knöll et al., 2013). As in the case of mislocalization errors, the functional significance of perisaccadic temporal distortions is also not well understood, but the phenomenon is interesting because it suggests that the mechanisms leading to perisaccadic spatial mislocalization may also influence other domains.

#### Forward and Convergent Remapping

Forward remapping, where neuronal RFs are presaccadically shifted by the upcoming saccade vector, has been suggested to provide a “preview” of stimuli that will appear in the RF after the saccade. This could allow forward remapping to be the neural basis of our robust visual perceptual stability (Duhamel et al., 1992). Some putative behavioral correlates of forward remapping have been explored. Using a free-viewing paradigm, Dorr and Bex (2013) found that observers report the location of a stimulus flashed 50–150 ms *before* saccade execution as being shifted in the direction of the impending saccade, as might be expected based on physiological descriptions of forward remapping (Duhamel et al., 1992). The perceived visual field location of tilt and motion adaptation aftereffects is also shifted in the forward remapping direction immediately before saccades (Melcher, 2007; Biber and Ilg, 2011), and subjects can experience acquisition of saccade targets before actual acquisition occurs (Hunt and Cavanagh, 2009). Most studies to date are based on the premise that forward remapping is the critical mechanism and typically probe only the forward remapping location; i.e., shifted in the same direction as the saccade. However, to assess the effects of convergent remapping it is really necessary to probe multiple visual field locations. This was recently done in a study by Zirnsak et al. (2011) where subjects fixated and adapted to a tilted grating in the periphery, and then made a tilt judgment about a test grating just before executing a saccade. The test grating could appear at either the forward remapping location (consistent with forward remapping) or partway between the adapting location and the saccade target (consistent with convergent remapping). Their findings were more consistent with convergent remapping. Importantly, behavioral experiments ostensibly observing perceptual consequences of remapping often assume that perceptual shifts associated with neuronal RFs shifting in a particular direction will be in the same direction, and while this may make intuitive sense, as we will see in “Functional Significance of Convergent Remapping” below, it is not necessarily the case.

### Neurophysiological Evidence of Remapping

In their seminal remapping study, Duhamel et al. (1992) reported that about half of LIP neurons exhibit forward remapping of their receptive fields. Between saccade target onset and saccade initiation, these neurons respond to visual stimuli appearing in their FF, that is, the visual field location the RF will occupy after the saccade is complete (see Figure 1). Physiological forward remapping has also been reported in many other visual areas, including SC (Walker et al., 1995; Churan et al., 2012), V2, V3 and V3A (Nakamura and Colby, 2002), FEF (both visual and visuomovement cells; Umeno and Goldberg, 1997, 2001) and MST (Inaba and Kawano, 2014); however, MT does not exhibit forward remapping (Ong and Bisley, 2011; Inaba and Kawano, 2014).

Typically only one visual probe stimulus is presented per trial in physiological studies, and often probe locations are drawn from a set of two: one in the classical RF and one in the FF. However, two notable studies probed locations on a two dimensional grid and provide a more complete picture of remapping in V4 (Tolias et al., 2001) and FEF (Zirnsak et al., 2014). In contrast to previous studies that reported forward remapping (at least in FEF), both of these studies found evidence of convergent remapping—the RF shifts towards saccade endpoints, not parallel to the saccade vector.

It remains an important open question whether convergent remapping reflects a neural mechanism that is distinctly different from forward remapping or if forward remapping is an aspect of convergent remapping not previously observed due to the use of limited stimulus and saccade configurations. That is, it is possible that a fortuitous geometric arrangement of visual probes (i.e., probes restricted to the classical RF and FF) led previous studies to conclude remapping was in the forward direction when it was actually convergent. To date, the only brain region where both forward and convergent mapping have been reported is FEF (Umeno and Goldberg, 1997, 2001; Zirnsak et al., 2014), suggesting that FEF might be a good target for careful examination of the differences between forward and convergent remapping. Zirnsak and colleagues present useful illustrations of how previous studies using stimuli only in the classical and forward remapping/FF locations could have mistaken convergent remapping for forward remapping (2014, Extended Data Figure 6). However, the main conclusion of their study was that the FEF *population average* exhibits convergent remapping. This finding is not inconsistent with an alternative interpretation where some FEF neurons remap convergently, while others remap in the forward direction, with the net result being that the population average remaps in the convergent direction. It is important to assess remapping at the level of both the single neuron, as well as at the population, in order to fully understand the underlying circuits. FEF has long been known to contain a variety of cell types including (but not limited to) visual neurons, which respond to visual stimulation only in the RF, and visuomotor neurons, which exhibit enhanced visual responses to RF stimuli when animals are preparing saccades towards the RF (Bruce and Goldberg, 1985). Since Zirnsak et al. (2014) pooled across these cell types, it is possible that some of what appears to be convergent remapping of visual responses has a pre-motor component, driven by an increased presaccadic visual response from visuomotor cells whose RFs happen to be located near the fixed, single saccade target. None of these details invalidate the general conclusion that a convergent process operates in FEF at the population level. However, additional studies will be required to assess the significance of convergent remapping in FEF and to definitively determine whether or not classical forward and convergent remapping reflect the same underlying neural circuit. There are several ways to address this question. Zirnsak and colleagues chose to increase the number of probe locations; however, to unambiguously prove convergent remapping, it is really necessary to show convergence occurs either in a significant fraction of single neurons or at the population level when averaged across visual neurons only. Alternatively, the use of multiple saccade vectors would help to dissociate between remapping of visual responses and some form of pre-motor activity.

### Integrating Behavioral and Neuronal Findings

Based on the phenomenological similarities between saccadic effects on visual perception and physiological remapping, it is tempting to conclude one is the consequence of the other. However, there are number of open questions that require resolution before we can safely make this conclusion.

#### Functional Significance of Convergent Remapping

Zirnsak et al. (2014) proposed that perisaccadic mislocalization errors are a consequence of neuronal convergent remapping. Remapping of fundamentally labeled-line neurons could lead to misattribution of remapped neuron activity to non-remapped locations in the visual field resulting in mislocalization. Clearly this would compromise localization accuracy, but it would also increase the number of neurons representing stimuli proximal to saccade endpoints, potentially decreasing detection thresholds and increasing saccade accuracy. An analogous effect has been demonstrated for spatial attention: attention can alter the structure of the spatial RF in several cortical areas (V4: Connor et al., 1996, 1997; MT: Womelsdorf et al., 2006, 2008; and LIP: Ben Hamed et al., 2002). These studies found that attention shifts RFs towards the attentional focus. However, attention results in a paradoxical perceptual repulsion, even though performance on detection and discrimination tasks is generally enhanced in the attended region (Suzuki and Cavanagh, 1997). Zirnsak and Moore (2014) further suggest that convergent remapping could even be related to automatic attention deployed at saccade endpoints. However, this model is at odds with some findings; specifically, while both saccades and attention do cause RF shifts towards the critical location (either saccade endpoint or attentional focus), their perceptual correlates appear to be in opposite directions, with mislocalizations towards and away from the critical location for saccades and attention respectively. This suggests that convergent remapping may not be mechanistically related to attentional deployment. Moreover, linking attentional deployment to convergent remapping may explain what triggers the remapping, but it does not speak to its functional significance. The functional role of increasing the number of neurons at an attended location is clear, but the importance of increasing the number of neurons representing a saccade target is less clear. Is it simply an epiphenomenal consequence of the obligatory link between the deployment of saccades and attention (to be discussed further in “Attentional Facilitation at Saccade Endpoints” below)? Or does it improve saccade targeting or visual stability in some way? Recent work has shown perisaccadic mislocalization errors occur in monkeys (Jeffries et al., 2007), which suggests a useful direction for future research; namely, to characterize the relationship between physiological RF shifts and behavioral mislocalization errors concurrently in the same experimental subjects.

#### Functional Significance of Forward Remapping

The functional significance of forward remapping is also not yet clear, although there has been considerable speculation that it plays an important role in perceptual stability during saccades. However, Churan et al. (2011) found that dense visual stimuli almost completely abolished remapping in SC, which raises questions about the function of remapping during natural vision, where the visual field is often filled with dense, cluttered stimuli. There also is evidence from physiological studies of LIP that remapping depends on attention (Gottlieb et al., 1998), so it could be that dense stimulus arrays make each individual component less salient or attention-grabbing and therefore less likely to trigger remapping. While this provides a plausible explanation for why remapping is restricted to sparse stimuli, it does not clarify the functional significance of remapping during natural vision.

Forward remapping could potentially provide a preview of the postsaccadic scene that can be used to detect scene changes occurring during the saccade. A bolder hypothesis is that forward remapping actually creates a transient spatiotopic representation in forward remapping neurons. In this model, the forward remapping mechanism instantiates the coordinate transformation necessary to convert retinotopic neurons into spatiotopic neurons based on a motor plan, and these neurons remain anchored in spatiotopic coordinates until the saccade is completed. After the saccade is complete, this spatiotopic representation decays back to retinotopic (elaborated in Burr and Morrone, 2011, 2012; Cicchini et al., 2013). Detailed characterization of the spatiotemporal dynamics of remapping to glimpse the actual map organization before, during, and after saccades could provide evidence for or against these models, but this has yet to be done. In addition, while recent work shows that neurons in FEF signal whether a change occurs in their RF across a saccade (Crapse and Sommer, 2012), it is not yet known whether this signal is dependent on forward remapping. It is also worth mentioning that functional roles of remapping other than visual stability have been proposed; for example, control of motor acts or maintenance of spatial memory (Bays and Husain, 2007).

There is little doubt that more work is needed to fully elaborate the relationship between physiological remapping and the multitude of effects saccades can have on visual perception. Understanding the functional significance of remapping will likely require investigations using more naturalistic viewing conditions, including denser stimulus displays that mimic the clutter and spatiotemporal complexity of natural vision. Although technically challenging, inactivation of the oculomotor CD signal, in combination with appropriate behavioral tasks and visual stimuli, could provide important new insight into how oculomotor commands interact with visual inputs during remapping and how these interactions perturb or stabilize perception. In addition, we submit that conclusions about the relationship between physiological and behavioral results are best supported by studies that concurrently examine both in the same subjects.

## Attentional Facilitation at Saccade Endpoints

“Premotor” theories of attention are premised on the idea that spatial attention reflects a planned but unexecuted saccade (Klein, 1980; Rizzolatti et al., 1987); enhanced behavioral performance attributed to “attention” in these models is the result of neuronal facilitation at the endpoint of a planned, but unexecuted, saccade and is generally believed to be instantiated by oculomotor circuits. Although difficult to prove, there is extensive circumstantial evidence in support of this idea. There is substantial overlap between brain regions activated by saccades and spatial attention (reviewed in Corbetta, 1998; Corbetta et al., 1998; Nobre et al., 2000; Perry and Zeki, 2000; Beauchamp et al., 2001), and psychophysical studies have established that involuntary attention-like benefits arise at saccade endpoints (Hoffman and Subramaniam, 1995; Kowler et al., 1995; Deubel and Schneider, 1996). There is little doubt that links exist between saccade planning and spatial attention; however, the extent and details of this relationship remain a matter of some debate. Specifically, although it is clear that single brain regions, like FEF, are involved in both saccade and attentional targeting, we do not yet know if individual neurons contribute to both processes.

### Behavioral Facilitation, Attention and Saccade Planning

If attention reflects covert saccade plans, then attention will be automatically deployed to every saccade endpoint. Conversely, attending to a location reflects an instantiated oculomotor plan, so saccades to attended locations should be faster or more accurate (Posner, 1980). Performance in many tasks is indeed enhanced at saccade endpoints (Chelazzi et al., 1995; Hoffman and Subramaniam, 1995; Kowler et al., 1995; Schneider and Deubel, 1995; Deubel and Schneider, 1996; Doré-Mazars et al., 2004; Montagnini and Castet, 2007; Rolfs and Carrasco, 2012; Zhao et al., 2012; Harrison et al., 2013), and saccadic reaction times (SRTs) are indeed shorter when making saccades to an attended location (Kowler et al., 1995). Neurological conditions that impair saccade targeting can also disrupt attentional control; for example, progressive supranuclear palsy, which impairs vertical eye movements, also impairs attentional deployment towards the upper visual field (Rafal et al., 1988). There is extensive evidence that saccade planning and attentional deployment circuits are anatomically overlapping (for example, see Fernandes et al., 2014). The crucial question moving forward is really whether the motor and attention parts of these circuits can operate independently. Simply put, is it possible to sustain attention across saccades, an ability critical for object tracking during natural vision?

There is recent evidence suggesting that exogenous or “bottom up” attention is more closely linked to saccade execution than endogenous or “top-down” attention. The temporal dynamics of behavioral facilitation at saccade endpoints more closely resemble the dynamics of exogenous attention; enhancement of orientation discrimination and contrast sensitivity are maximal 100–150 ms after saccades are cued (Castet et al., 2006; Rolfs and Carrasco, 2012), which is similar to the time required to deploy exogenous attention (Murphy and Eriksen, 1987) but less than the time needed to deploy endogenous attention (Liu et al., 2007). Similarly, SRTs to attention-capturing stimuli are decreased just after stimulus onset, but this effect disappears by 600 ms, which is similar to the pattern of non-saccade RTs observed when attention is exogenously captured (Crawford and Muller, 1992). In a case study Smith et al. (2004) described a patient with a congenital oculomotor paralysis who also showed impaired saccade targeting exhibited deficits in exogenous, but not endogenous, attentional orienting, which suggests an anatomical distinction between saccade targeting and top-down attention. In addition, when considering links between saccade execution and attentional deployment, it is crucial to distinguish between shifting attention from one location to another and sustaining an existing locus of attention. It is possible that shifting, but not sustaining, attention depends on oculomotor circuits; consistent with this idea is the finding that sustaining attention can actually increase SRTs in some paradigms (Belopolsky and Theeuwes, 2009, 2012). Golomb et al. (2008) showed that attentional benefits are detectable at non-endpoint locations tens of milliseconds after saccades, suggesting that even if attentional resources are momentarily diverted to the saccade endpoint, they can be quickly redirected to maintain a locus of attention. Taken together, these results provide convincing evidence that the relationship between attention and saccades, while significant, is sensitive to nuances of task design and the methods used to guide attentional deployment. Specifically, it is possible to sustain top-down attention to a non-fixational location through saccades, despite the automatic deployment of attention to saccade endpoints.

### Neuronal Saccade Target Enhancement

It is well established that covert attention can facilitate neuronal responses to attended stimuli (Moran and Desimone, 1985; Motter, 1993; Treue and Maunsell, 1996; Roelfsema et al., 1998; Ito and Gilbert, 1999; McAdams and Maunsell, 1999; Reynolds et al., 2000). Fischer and Boch (1981) first described a similar, attention-like presaccadic facilitation of visual responses in V4 neurons just before saccades into the RF. Similar findings have been reported in SC (Li and Basso, 2008), V1 (Supèr et al., 2004), LIP (Colby et al., 1996; Ipata et al., 2006), FEF (Schall and Hanes, 1993) and IT (Chelazzi et al., 1993; Sheinberg and Logothetis, 2001). The relationship between saccade endpoint facilitation and attentional facilitation has arguably been best studied to date in V4, where planning a saccade into the RF of a neuron has been shown to modulate response variability (Moore and Chang, 2009; Steinmetz and Moore, 2010), contrast and luminance sensitivity (Han et al., 2009), and even popout effects (Burrows and Moore, 2009). These neurophysiological findings are consistent with behavioral work showing obligatory attentional deployment at saccade endpoints. In brain regions where electrical stimulation can evoke saccades, sub-threshold stimulation (i.e., too weak to elicit a saccade) can produce attention-like behavioral benefits at would-be saccade endpoints (Moore and Fallah, 2001, 2004; Cutrell and Marrocco, 2002; Müller et al., 2005; Armstrong and Moore, 2007). In FEF, sub-threshold microstimulation results in attention-like modulation of visual responses in V4 neurons, presumably via feedback projections (Moore and Armstrong, 2003; Armstrong et al., 2006).

While data from microstimulation experiments are generally consistent with premotor theories, it is important to note that microstimulation activates all cell types, and even at the low current levels used in these studies, likely activates a large and heterogeneous population of neurons. As a result, these studies serve only to confirm there are both cells that contribute to saccade planning and cells that contribute to attentional targeting in a given area, but are insufficient to prove that single cells contribute to both. In fact, Thompson et al. (2005) showed that in FEF, visual neurons are facilitated by attention while movement neurons are more likely to be inhibited. In humans some parietal lesions impair saccade execution without affecting attentional targeting and *vice versa*, suggesting at least a coarse anatomical segregation between the two processes (Blangero et al., 2010). Murthy et al. (2001), trained monkeys to prepare a saccade to one location and on some trials, changed the target location just before saccade initiation. On these redirection trials they observed attention-like facilitation in FEF neurons when the redirected target appeared inside the neuron’s RF, even when the animal failed to redirect. This finding is important since it demonstrates attention-like facilitation at a non-endpoint location just before saccades, indicating that while physiological attention does automatically deploy to saccade endpoints, it may also deploy to non-endpoint locations at the same time.

### Premotor Theories: Conclusions

The critical question posed by premotor theories of attention is whether or not covert attention toward a visual field location is anything more (or less) than planning, but not executing, a saccade towards the “attended” location. At the physiological level we can ask two questions: (1) do single neurons instantiate both saccades and attention? and (2) if single brain regions instantiate both saccades and attention, is that because every neuron does both or because there is a heterogeneous neuronal population with some neurons planning saccades and others targeting attention? There is clearly evidence of the latter from both physiological recording and microstimulation studies. These studies, though informative, have been insufficient to definitively confirm the former. Moreover, as noted above, some data suggest the answers to these questions may critically depend on the specific form of attention under study, e.g., endogenous attention seems more readily dissociable from saccades that exogenous attention (Crawford and Muller, 1992; Smith et al., 2004), but this distinction has not yet to be carefully explored using neurophysiological methods.

## Saccades and attention at Non-Endpoint Locations

Whether or not saccades and attention are fully dissociable, it is clear we can direct and sustain attention towards environmental features other than saccade targets while making frequent saccades (Kowler et al., 1995; Montagnini and Castet, 2007; Golomb et al., 2008; Jonikaitis et al., 2013; Lisi et al., 2015). This is a critical precondition if attention is to play an important functional role during natural vision. In primates, saccades and attention are likely to be directed towards different locations when performing complex behaviors, e.g., during social interactions, when making visually guided reaches, etc. Therefore, it is important to understand how attention is sustained across eye movements. Although attentional stability has been less studied than visual stability, several recent studies have explored attentional stability and possible links between visual and attentional stability.

### Perisaccadic Behavioral Findings

**Figure 3:**
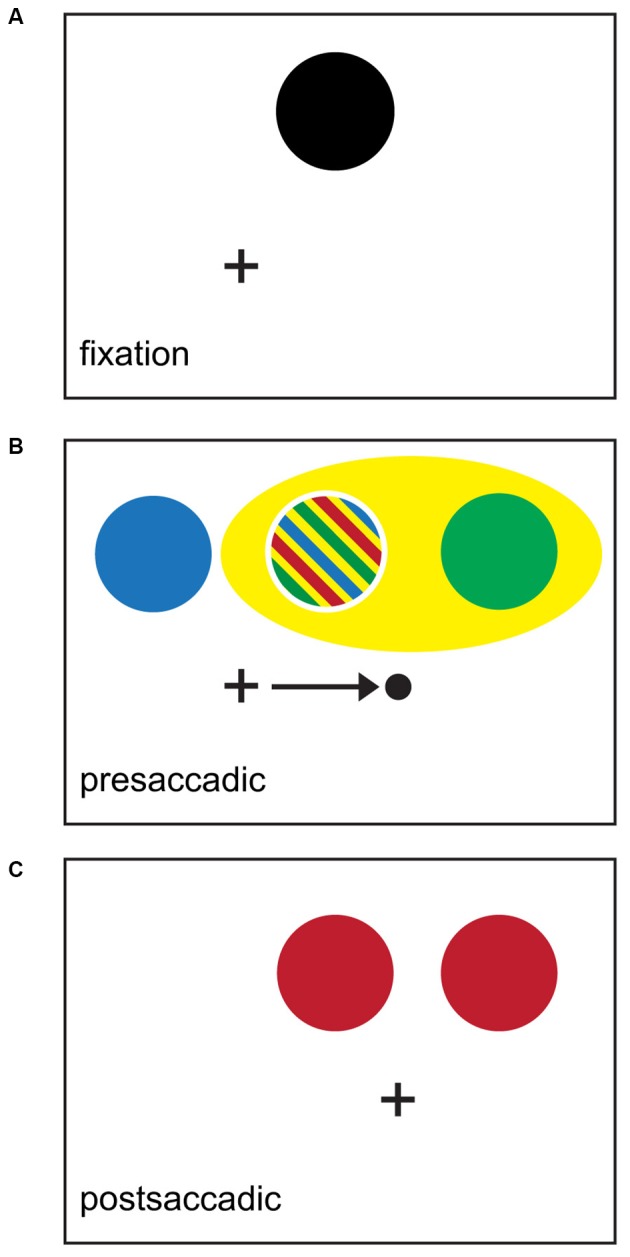
**Schematic summary of perisaccadic attentional effects.** Black crosses indicate point of initial fixation; black dot and arrow indicate saccade targets and vector respectively. **(A)** Black area indicates locus of spatiotopic attention triggered from an endogenous or exogenous source. Subjects must sustain attention at this point in a spatiotopic reference frame across saccade to correctly perform the task. **(B)** Presaccadic epoch. Colors indicate regions where attentional benefits have been demonstrated experimentally. Predictive attentional targeting is shown in blue (Rolfs et al., 2011). Shifting attention towards the FF location is shown in green (Mathôt and Theeuwes, 2010a), and spreading attention encompassing both the attentional locus and the FF location in yellow (Mathôt and Theeuwes, 2010a). Striped region indicates that attentional benefit was found in multiple studies. **(C)** Postsaccadic epoch. Location of the retinotopic attentional trace (RAT) is shown in red (Posner and Cohen, 1984; Golomb et al., 2008).

Several distinct regions of attentional facilitation arise around the time of a saccade during attentional cueing tasks, including some at nominally task irrelevant locations (Figure 3). In one of the earliest studies of spatiotopic attention, Posner and Cohen (1984) showed persistent postsaccadic attentional enhancement, i.e., decreased RTs when responding to targets appearing at attended locations, at the retinotopic location of exogenous cues. More recently, Golomb et al. (2008) showed a similar persistence of attention in a retinotopic reference frame using an endogenous attention paradigm in which subjects were instructed to remember a location in a spatiotopic reference frame and make a speeded orientation judgment about targets most likely to appear at the remembered spatiotopic location. In their task design, once the saccade was completed the retinotopic location of the cue was no longer relevant, since it did not correspond to a likely target location. Nonetheless, they observed significant postsaccadic enhancement at the retinotopic location. This involuntary facilitation at presaccadically attended but postsaccadically task-irrelevant locations, known as the retinotopic attentional trace (RAT), is spatially distinct and temporally concurrent with facilitation at the task-relevant spatiotopic location (Golomb et al., 2011). The RAT has also been demonstrated using exogenous attention (Jonikaitis et al., 2013) and in a statistical learning paradigm (Jiang and Swallow, 2013). These studies provide compelling evidence that the native reference frame for attention is retinotopic and are consistent with a model in which a fundamentally retinotopic attentional representation in the brain is actively updated around the time of saccade execution to compensate for changes in eye position (Golomb et al., 2008).

Attentional benefits at nominally unattended locations are also detectable before saccades. When subjects are instructed to sustain attention in a spatiotopic reference frame, a predictive attentional focus develops at the retinotopic location where the attended location will fall once the saccade is complete. This appears as presaccadic enhancement of responses to targets appearing shifted in the *anti-saccade direction* from the attended location (see Figure 3). This was first described by Rolfs et al. (2011) in a study that used a double step saccade task to simultaneously instruct saccades and implicitly deploy attention. Rolfs et al.’s findings suggest that attention is engaged predictively before saccade onset to ensure an accurate attentional topography is already in place when the eyes land at the saccade endpoint, presumably to minimize or even eliminate the time when attention is mistargeted at the onset of the next fixation (at the cost of a brief period of presaccadic attentional mislocalization). We will use the term *predictive attentional targeting* to refer to this location-specific presaccadic finding. Predictive attentional targeting has been demonstrated under a number of conditions: when attention is deployed in response to exogenous cues (Jonikaitis et al., 2013), in a visual masking paradigm (Hunt and Cavanagh, 2011) and during attentive tracking of moving stimuli (Szinte et al., 2015).

On the other hand, Mathôt and Theeuwes (2010a) showed that, in addition to predictive attentional targeting, presaccadic attentional benefits are also observed at a location shifted from the initial attended location in the pro-saccade direction. This location corresponds to the physiological FF of the attended RF as described in “Integrating Behavioral and Neuronal Findings” below. However, a recent study suggests that these pro-saccadic anticipatory shifts may be better described as attentional spreading in the pro-saccade direction (Harrison et al., 2012), as opposed to the shift of a spatially well-localized attentional focus, as is observed during the postsaccadic period (Golomb et al., 2011).

### Neurophysiological Findings

Functional MRI studies of humans performing a spatiotopic attention task have found patterns of neural attentional modulation consistent with the RAT in a number of retinotopically organized cortical visual areas, including areas V1, V2 and V4 (Golomb et al., 2010). Similarly, postsaccadic modulation of the N1 (Golomb et al., 2010) and P1 (Talsma et al., 2013) ERP components in occipital electrodes is also consistent with the RAT when subjects must sustain a spatiotopic attentional locus across saccades.

There are only a few reports suggesting that monkeys can deploy attention in both spatiotopic and retinotopic reference frames (Rawley and Constantinidis, 2010; Marino and Mazer, 2015). Rawley and Constantinidis (2010) demonstrated that individual neurons in parietal area 7a can show attentional modulation when the animal deploys attention in either reference frame. However, because the stimuli in that experiment usually appeared >200 ms after the saccade offset, it is impossible to determine whether 7a neurons exhibit correlates of the RAT, which decays to baseline levels with a time constant of ~150 ms (Golomb et al., 2008) from the time of saccade onset. Also, Rawley and Constantinidis (2010) studied each neuron under either spatiotopic or retinotopic task conditions, but not both; so it is not known whether individual 7a neurons switch reference frames, or if there are two neuronal subpopulations with differing reference frame coding. In LIP, there is evidence neurons signal the attentional priority of stimuli that will be brought into the RF by an upcoming saccade (Mirpour and Bisley, 2012). Similar findings have been reported in FEF, where responses to forward remapping stimuli reflect stimulus salience (Joiner et al., 2011). Finally, Khayat et al. (2004) showed that the latency of attentional modulation in V1 is reduced when saccades bring stimuli into the RF compared to when stimuli appear in the RF without a saccade, suggesting that even as early as V1 information about attentional state can update in preparation for saccades.

### Integrating Behavioral and Neuronal Findings

Presaccadic predictive attentional targeting (Rolfs et al., 2011) and the postsaccadic RAT (Golomb et al., 2008) provide key anchor points that can inform our cognitive models and guide our neurophysiological investigations into the neural implementations of transsaccadic attentional targeting. At the behavioral level, anticipatory or predictive attentional shifts make functional sense—these shifts allow the visual system to be in the correct attentional state immediately after a potentially disruptive saccade; without a predictive component, attention could lag behind feedforward perceptual processing. The consequence of this lag would be a period of attentional misallocation. The time course of the RAT reflects the switching time, or at least the switching-off time, associated with allocating attention to a new location in the visual field. Somewhat paradoxically, the combination of a predictive mechanism and a slow off-time results in a period of split attention, where attentional facilitation is detectable at least two distinct locations in the visual field (Golomb et al., 2008, 2011). The transient appearance of multiple attentional loci could contribute to perisaccadic mislocalization.

#### Remapping RFs vs. Shifting Attentional Pointers

Cavanagh et al. (2010) proposed that physiological forward remapping of neuronal RFs may be an incidental side-effect of a transfer of information between neurons whose primary function is to instantiate presaccadic predictive attentional targeting, or, in their terminology, the updating of attentional pointers. The authors suggest that this remapping or transfer of attentional state, in addition to (or instead of) remapping of spatial or featural selectivity could account for Rolfs et al. (2011) finding that attentional benefits update predictively before saccade initiation.

Although predictive attentional targeting and forward remapping of spatial selectivity appear similar, it is not at all clear that forward remapping can be characterized as an incidental effect that occurs during the anticipatory updating of attentional pointers or is even involved in predictive attentional targeting (Mayo and Sommer, 2010; Melcher, 2010). First, we currently know little about how or even if neuronal attentional modulation remaps in preparation for saccades or even if it remaps at all. Neurophysiological studies to date have focused almost exclusively on perisaccadic changes in visual spatial selectivity and not modulation state; such studies provide strong support for transfer of information about stimulus location (Duhamel et al., 1992; Walker et al., 1995; Umeno and Goldberg, 2001; Nakamura and Colby, 2002) and features (Subramanian and Colby, 2014) between neurons in anticipation of saccades. The limited studies that have examined attentional updating in neurons reported predictive effects in LIP (Mirpour and Bisley, 2012) and FEF (Joiner et al., 2011); however, because complete spatial RF profiles were not characterized in these reports, the results do not address the relationship between attentional updating and RF remapping (Joiner et al., 2011).

**Figure 4:**
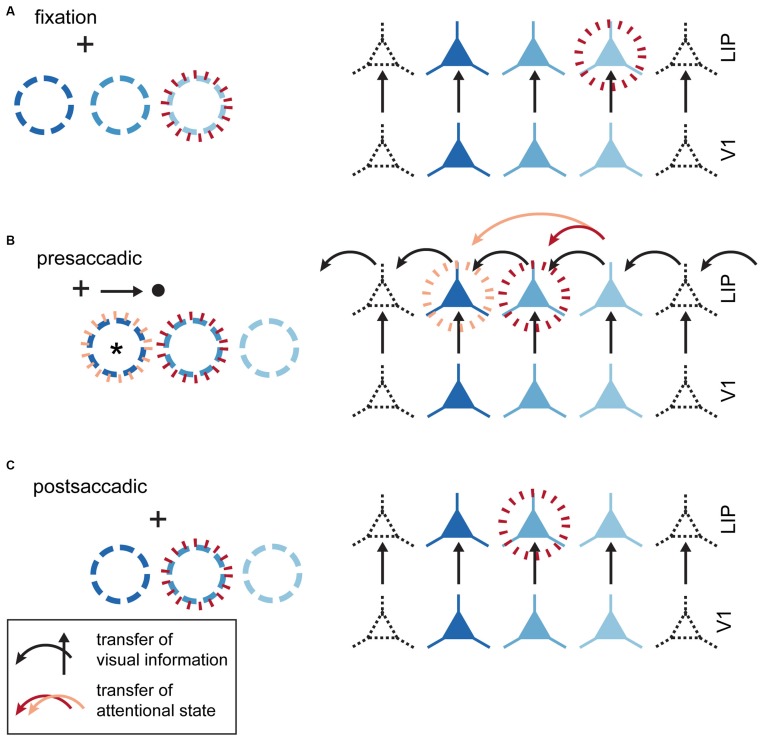
**Visual and attentional remapping. (A)** Fixation epoch (prior to saccade plan instantiation). Circles in the left column indicate the position of neuronal RFs; neurons in the right column are colored to indicate corresponding RFs in the left column. Red dashed circles indicate the location of the attentional locus and associated facilitated neurons. **(B)** Presaccadic epoch (saccade plan instantiated, but not initiated). Black arrows indicate “horizontal” transfer for visual information between neurons triggered by the saccade plan. These transfers are not necessarily instantiated by horizontal connections in cortex, but could equally well reflect changes in the pattern of feedfoward input. The effect of this horizontal transfer is to shift spatial RFs to the right in response to planning a saccade to the right. Two different attentional updating mechanisms are illustrated. (1) Red arrow represents transfer of attentional facilitation as part of the visual remapping signal, which would result in attentional facilitation at the originally attended location (red dashed circle). This is inconsistent with predictive effects demonstrated by Rolfs et al. (2011). (2) Orange arrow: Attentional and visual signals are remapped separately. To generate the observed predictive attentional shifts, attentional state must be remapped twice as far as the visual signal. Importantly, this neuron will not represent the attended location after the saccade is executed, so such a signal would not be useful for stabilizing spatiotopic attention across the saccade. **(C)** Postsaccadic epoch. RFs revert to their original retinotopic location with attention deployed to the correct spatiotopic location.

Cavanagh et al. (2010) suggested that RF remapping reflects an active horizontal transfer of information between neurons that makes the RF appear to shift in the pro-saccade direction (although the information transfer is actually in the anti-saccade direction; see Figure 4). While the characterization of remapping as information transfer, rather than spatial RF remapping, may be a useful formulation, there is not yet sufficient data to warrant abandoning the idea that updating of a RF location is an integral and potentially functionally significant perisaccadic effect. The crucial question is: exactly what information is transferred presaccadically? Does the transfer include attentional state, feature selectivity, spatial selectivity, etc.? As illustrated in Figure 4, it is not possible to transfer both attentional state and spatial selectivity while still accounting for the predictive attentional targeting behavioral results of Rolfs et al. (2011). At this point, numerous neurophysiological studies have shown that in many areas spatial RFs do undergo spatial remapping during the same presaccadic interval in which attentional pointers are thought to predictively remap. If the transfer of information serves primarily to update attentional pointers but also incidentally updates spatial selectivity, attention would be transferred to the *remapped* spatial location, resulting in no net change in the environmental location of the attentional focus in the presaccadic period. Simply put, if there is horizontal transfer of attentional state *and* forward remapping in the same neuronal population, the attentional focus will not shift to the predictive location and but instead be stabilized at the spatiotopic location, which would actually be functionally advantageous. This would effectively stabilize the attentional topography across the saccade rather than lead to predictive attentional targeting (see Figure 4). In order to simultaneously account for both observed predictive psychophysical effects and observed physiological forward remapping there must be some degree of dissociation between the transfer of attentional state and spatial selectivity.

## Open Questions and Future Directions

There now exists a substantial body of behavioral and neurophysiological work related to perisaccadic changes in visual perception and visual attention. Reviewing the breadth of this work makes it clear substantial progress has been made by the field in recent years; however, there remain significant discrepancies or apparent inconsistencies in the experimental data where further work is required. For example, it is critical to determine whether apparent differences between spatial RF remapping (i.e., forward vs. convergent remapping) are due to functional differences between cells and brain areas or reflect methodological differences perhaps related to task details, stimulus properties, or even neurophysiological recording methods.

There are also important fundamental questions that remain unanswered. Of the many perceptual phenomena linked to saccades discussed here, e.g., SSD, mislocalization errors, presaccadic and postsaccadic shifts in aftereffect localization, and changes in attentional topography, which are really directly linked to physiological RF remapping? Are visual and attentional stability instantiated by the same or different neural circuits? In addition, while it is tempting to link perisaccadic behavioral findings to perisaccadic neurophysiological findings—for example, predictive updating of attentional topography and forward RF remapping or behavioral evidence of spatial compression and convergent remapping—no studies to date have actually observed these behavioral findings and neurophysiological findings concurrently. Observing physiology or behavior on their own are useful exploratory steps but are not sufficient to demonstrate either sufficiency or necessity. To better understand the relationship between these neurophysiological and behavioral phenomena, it is crucial undertake the difficult process of studying both behavioral and physiological effects in the same animals, at the same time, and to the extent it is possible, in the context of natural, dynamic visual stimuli.

## Author Contributions

ACM and JAM prepared manuscript together.

## Conflict of Interest Statement

The authors declare that the research was conducted in the absence of any commercial or financial relationships that could be construed as a potential conflict of interest.
